# Modeling rebound bone loss following denosumab discontinuation and sequential zoledronate therapy in TgRANKL osteoporotic mice

**DOI:** 10.3389/fendo.2026.1783656

**Published:** 2026-02-26

**Authors:** Vagelis Rinotas, Eleftheria-Dimitra Ntouskou, Melina Dragolia, Vasileios Ntafis, Maria P. Yavropoulou, Athanasios D. Anastasilakis, Polyzois Makras, Eleni Douni

**Affiliations:** 1 Institute for Bioinnovation, Biomedical Sciences Research Center “Alexander Fleming”, Vari, Greece; 2 Institute for Fundamental Biomedical Research, Biomedical Sciences Research Center “Alexander Fleming”, Vari, Greece; 3 Endocrinology Unit, 1st Department of Propaedeutic and Internal Medicine, Medical School, National and Kapodistrian University of Athens, Athens, Greece; 4 Department of Endocrinology, Diabetes and Metabolism, 424 General Military Hospital, Thessaloniki, Greece; 5 Department of Endocrinology and Diabetes and Department of Medical Research, 251 Hellenic Air Force, Veterans Affairs General Hospital, Athens, Greece; 6 Laboratory of Genetics, Department of Biotechnology, Agricultural University of Athens, Athens, Greece

**Keywords:** animal models, bone marrow adiposity, denosumab discontinuation, osteoclasts, osteoporosis, RANKL, zoledronate

## Abstract

**Background:**

Receptor activator of nuclear factor-κB ligand (RANKL) plays a central role in regulating osteoclast formation and bone resorption, while its inhibition by the monoclonal antibody denosumab serves as an effective antiresorptive treatment for postmenopausal osteoporosis. However, denosumab discontinuation triggers a severe rebound effect involving rapid bone mineral density (BMD) loss, accompanied by an overshooting of bone turnover markers (BTMs), and increased risk of multiple fractures. Preclinical studies investigating this rebound phenomenon after denosumab discontinuation have been limited, mainly because denosumab does not cross-react with murine RANKL. This study explores the rebound phenomenon in a transgenic mouse model of osteoporosis expressing human RANKL (TgRANKL) and evaluates the impact of sequential zoledronate therapy.

**Methods:**

TgRANKL mice were divided into four experimental groups: vehicle control, continuous denosumab treatment, denosumab withdrawal, and sequential denosumab followed by zoledronate, including an additional follow-up phase after zoledronate discontinuation. Skeletal alterations were characterized using microCT, histomorphometric assessments, serum bone turnover markers (BTMs), and bone gene expression analyses.

**Results:**

Denosumab therapy rescued the osteoporotic phenotype of TgRANKL mice, whereas its discontinuation resulted in a rebound bone loss accompanied by elevated bone turnover markers. Denosumab also inhibited bone marrow adipose tissue formation in TgRANKL mice, while its discontinuation led to moderate reformation of marrow adiposity. Sequential administration of zoledronate effectively prevented the rebound bone loss response. However, discontinued therapy after denosumab-zoledronate sequence, showed that the protective effects of zoledronate were not persistent.

**Conclusions:**

Our findings establish TgRANKL mice as a unique osteoporotic model for investigating the mechanisms driving denosumab rebound and testing sequential antiresorptive strategies.

## 1 Introduction

Receptor activator of nuclear factor-κB ligand (RANKL), constitutes the master regulator of osteoclast differentiation and activation through binding to its cognate RANK receptor (1). Osteoprotegerin (OPG) acts as a decoy receptor for RANKL, inhibiting its ability to activate osteoclasts (2). Therefore, the RANKL/OPG ratio is critical for maintaining physiological bone density and structure, while elevated RANKL/OPG ratio is observed in conditions characterized by increased bone turnover such as osteoporosis (3) which is characterized by low bone mineral density (BMD), significant bone loss and increased risk of fractures (4). The inhibition of RANKL leads to increased bone mass by reducing osteoclastogenesis and bone resorption. Denosumab, a fully human monoclonal antibody that targets human RANKL (huRANKL) prevents the activation of RANK signaling and thereby the maturation and activation of osteoclasts (5). Subcutaneous administration of denosumab at 60 mg every 6 months is recommended for the treatment of postmenopausal osteoporosis (6). The FREEDOM trial and its extension studies have shown that continuous denosumab treatment for up to 10 years leads to progressive increases in BMD at multiple skeletal sites, further reducing the risk of vertebral and non-vertebral fractures without increased risks of infection, immunogenicity or cancer (7). However, discontinuation of denosumab therapy results in rapid BMD loss and increased risk of multiple vertebral fractures within 12 months after the last injection, referred as rebound bone loss, that is associated with an increase of bone turnover markers above pretreatment levels probably due to enhanced osteoclastogenesis (8, 9). To prevent rebound resorption following denosumab discontinuation, it is currently recommended a switch to another antiresorptive agent. Various strategies and treatments have been explored including administration of zoledronate to preserve bone structure after denosumab discontinuation (10, 11).

The mechanisms responsible for rebound resorption after denosumab discontinuation remain largely unknown, highlighting the need for preclinical investigations. Ongoing research is needed to better understand the mechanisms that cause rebound BMD loss and to evaluate therapies following denosumab discontinuation. However, relevant preclinical studies in mouse models are limited mainly because denosumab is not effective in mice since it does not bind to murine RANKL due to species specificity (12). Therefore, denosumab discontinuation is mainly indirectly modeled in non-osteoporotic baseline conditions by administration of mouse RANKL inhibitors such as OPG-Fc or monoclonal antibodies (13–16).

We previously developed transgenic mice expressing huRANKL by introducing a 200 kb genomic fragment containing both the coding sequence and regulatory elements of the *huRANKL* gene (17). This approach yielded a physiologically relevant expression pattern that closely mimics the endogenous expression of the mouse *Rankl* gene. In these TgRANKL mice, huRANKL overexpression results in elevated bone turnover, trabecular bone loss, increased cortical porosity, and abnormal accumulation of bone marrow adiposity. In this study, we established a murine model to examine rebound bone resorption after denosumab withdrawal by treating osteoporotic TgRANKL mice. The study assessed the therapeutic efficacy of denosumab, determined the extent of bone loss following its withdrawal, and evaluated the effects of subsequent zoledronate administration. *In vivo* imaging was employed to monitor treatment outcomes and rebound phenomena throughout the experimental period. At the study endpoint, femoral bones were examined by micro-computed tomography (microCT) and histological analysis, while bone turnover markers were measured in both femoral tissue and serum to link biochemical changes with structural alterations. This integrative approach enabled a thorough evaluation of treatment responses and rebound effects in a clinically relevant preclinical model.

## 2 Materials and methods

### 2.1 Mouse husbandry

Osteoporotic TgRANKL mice (Tg5519 line) (17, 18), and WT control mice of C57BL/6J background were maintained and bred in the animal facility of Biomedical Sciences Research Center “Alexander Fleming” under specific pathogen-free conditions. All animal work was approved by the Institutional Protocol Evaluation Committee and was licensed by the Veterinary Authorities of Attica Prefecture (registered code: 203222/11-03-2020) in compliance with the PD 56/2013 and the European Directive 2010/63/EU.

### 2.2 Treatment protocol

**Figure 1:**
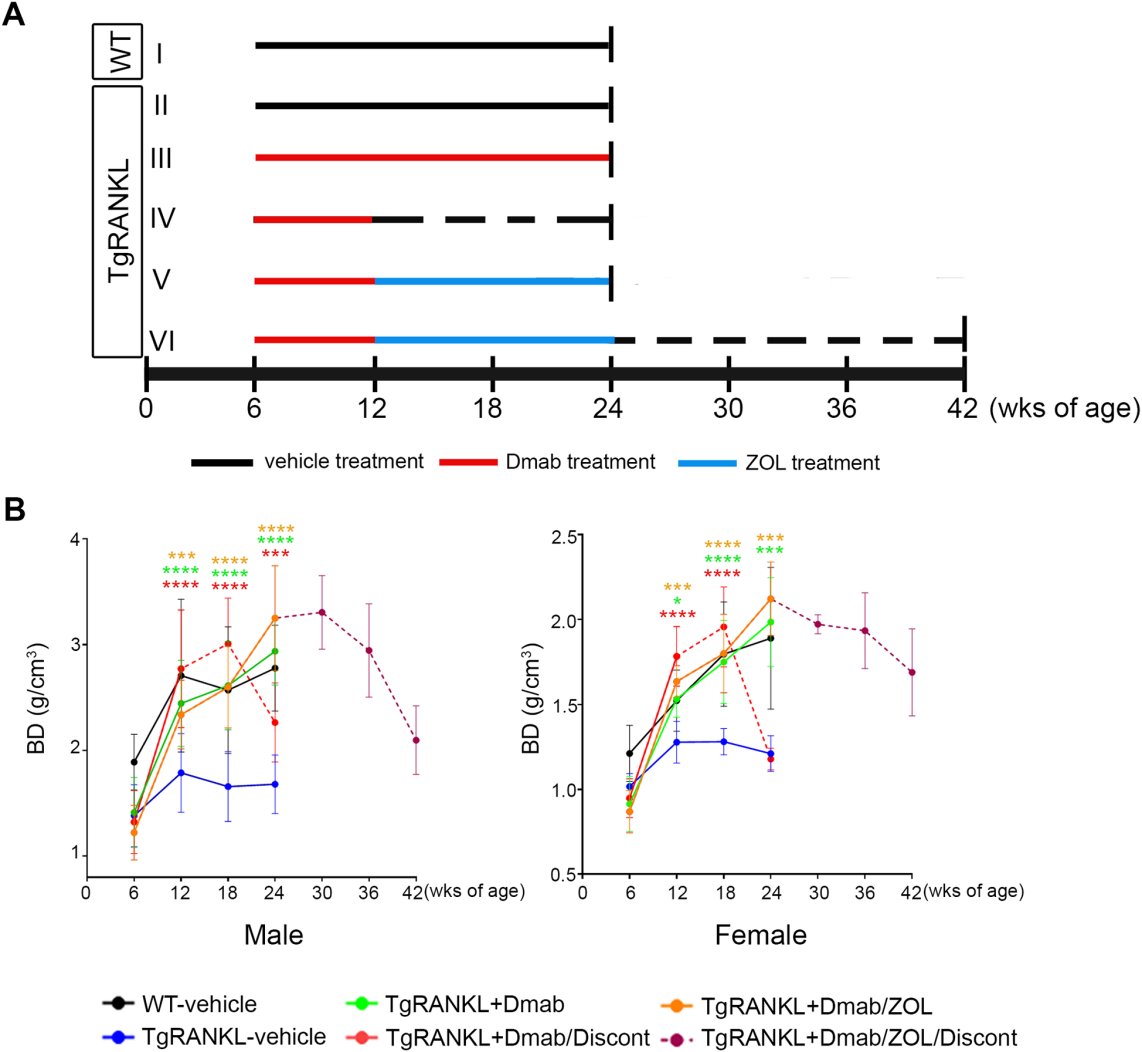
Schematic overview of the experimental design and *in vivo* assessment of bone density throughout the study. **(A)** WT and TgRANKL mice were divided into 6 treatment groups (n=9–12 per group, both sexes) with equal average body weights: I: WT-vehicle group, II: TgRANKL vehicle group, III: TgRANKL+Dmab group, IV: TgRANKL+Dmab/Discontinuation group, V: TgRANKL+Dmab/ZOL group and VI: TgRANKL+Dmab/ZOL/Discontinuation group. Dotted lines indicate treatment discontinuation. All mice were sacrificed 12 weeks after denosumab (Dmab) discontinuation except for half of the mice from group VI which were sacrificed 18 weeks after ZOL discontinuation. **(B)** Imaging and acquisition of bone density from distal femurs among experimental groups during the study, BD: Bone Density (g/cm^3^). Data represent mean values ± SD. Statistical analysis using two-way ANOVA followed by Tukey’s *post-hoc* test was performed exclusively on data from TgRANKL groups collected between weeks 6 and 24 of age. Red, green and orange asterisks correspond to color-assigned groups and indicate the statistical significance compared to TgRANKL-vehicle group, respectively. *P<0.05, ***P<0.001, ****P<0.0001.

A schematic of the study design is shown in **Figure 1**. 6-week-old WT and TgRANKL mice with equal average body weights were divided into 6 treatment groups. Group I: WT vehicle group injected subcutaneously (s.c.) with phosphate-buffer saline (PBS) twice per week for 18 weeks (n=10, both sexes), Group II: TgRANKL vehicle group injected s.c. with PBS twice per week for 18 weeks (n=10, both sexes), Group III: TgRANKL+Dmab group injected s.c. with 3 mg/kg denosumab (Prolia^®^) once per week for 18 weeks (n=10, both sexes), Group IV: TgRANKL+Dmab/Discontinuation group injected s.c. with 3 mg/kg denosumab once per week for 6 weeks followed by PBS injections s.c. for 12 weeks (n=11, both sexes), Group V: TgRANKL+Dmab/ZOL group injected s.c. with 3 mg/kg denosumab once per week for 6 weeks followed by intraperitoneal (i.p.) injections of 150 μg/kg zoledronate (ZOL) (Aclasta^®^) twice per week for 12 weeks (n=7, both sexes) and Group VI: TgRANKL+Dmab/ZOL/Discontinuation group injected s.c with 3 mg/kg denosumab once per week for 6 weeks then switch to i.p. injections of 150 μg/kg zoledronate twice per week for 12 weeks and cessation of zoledronate for another 18 weeks (n=5, 3 males and 2 females). Body weight was recorded weekly for each mouse. All mice were euthanized 12 weeks after denosumab discontinuation except the mice from Group VI experimental group which were euthanized 18 weeks after ZOL cessation. Upon sacrifice, both femurs were harvested from all animals. Right femurs underwent initial ex vivo microCT scanning, followed by either random allocation for osmium tetroxide staining and bone marrow adipose tissue (BMAT) quantification (n=5-6) or decalcification and embedding for histological evaluation (n=5) with balanced sex representation. Left femurs were snap-frozen and stored at -80°C for RNA extraction and gene expression analysis (n=4/group, sex-balanced) or reserved for additional assessments. In addition, blood was collected to measure serum Ctx, P1NP and free calcium. Mice were not fasted prior to blood collection. Euthanasia was performed with CO_2_ with a 30% per minute displacement of the chamber volume, followed by cervical dislocation (Directive 2010/63/EU).

### 2.3 *In vivo* X-ray imaging and acquisition of bone density

Planar X-ray images of the animals were acquired using the *In vivo* Xtreme imaging system (Bruker). Mice were anesthetized with isoflurane in an induction chamber ( 2 % isoflurane in ${\text{O}}_{2}$ at 5 L/min), transferred to the *In-Vivo* Xtreme imaging cabinet, and maintained under anesthesia during acquisition ( 2% isoflurane in ${\text{O}}_{2}$ at 1 L/min), with dexpanthenol ophthalmic gel applied for corneal protection. The Bone Density Software (Bruker Molecular Imaging Software) was used to measure the bone density (BD) (g/cm^3^) in order to monitor disease development. Bone density software Module utilizes low-dose “soft” X-rays to accurately measure bone density at the midshaft region of the distal femur.

### 2.4 Histopathological analysis

Femurs were fixed in 10% formalin (Carlo Erba) for 16 hours, decalcified in 13% EDTA, and embedded in paraffin. Sections of 5‐μm thickness were stained with hematoxylin/eosin. Osteoclasts were visualized by staining for TRAP activity using a leukocyte acid phosphatase kit (Sigma‐Aldrich). TRAP staining was quantified as total osteoclast number in total bone surface and as an osteoclast surface fraction (percentage of osteoclast surface in total bone surface, Oc.S/BS, %). Analysis was focused on the endocortical bone surfaces at the metaphyseal region of distal femur and was performed using the open source software for bone histomorphometry “TrapHisto” (19).

### 2.5 MicroCT analysis

Femurs and lumbar spine were fixed in 10% formalin overnight at 4°C and then washed and stored in PBS. Microarchitecture of the distal femurs and lumbar spine (L5) from all experimental mice was evaluated using a high-resolution SkyScan1172 microtomographic (microCT) imaging system (Bruker). Images were acquired at 50 KeV, 100 µA with a 0.5 mm aluminum filter. Three-dimensional reconstructions (8.8 mm cubic resolution) were generated using NRecon software (Version 1.7.4.2, Bruker) as previously described (17, 18). The trabecular area of the distal femur and lumbar spine was assessed through bone volume fraction (BV/TV, %), bone volume (BV, mm^3^), trabecular number (Tb.N, mm^-1^), trabecular separation (Tb.S, mm), connectivity density (Conn.Dn, mm^-3^) and bone mineral density (BMD, g/cm^3^). Femoral trabecular geometry was assessed using 300 continuous CT slides (1800 µm) located at the trabecular area 50 slides underneath the growth plate and the lumbar spine’s trabecular geometry was assessed using 250 continuous CT slides (1487 μm). Femoral cortical geometry was assessed using 100 continuous CT slides (600 µm) located at the femoral midshaft as previously described (17, 20). Cortical bone was assessed through cortical bone volume fraction (Ct.BV/TV, %), cortical thickness (Ct.Th, mm), bone marrow volume (BMV, mm^3^), total cortical porosity (Po(tot), %) and tissue mineral density (TMD, g/cm^3^).

### 2.6 Quantitation of bone marrow adipose tissue by Osmium Tetroxide staining and microCT

Femurs were stained with osmium tetroxide for analysis of BMAT, as previously reported (21, 22) with optimized modifications. Briefly, upon microCT scanning of bone samples, femurs were decalcified in 13% EDTA, pH 7.4, for 14 days. After washing with tap water for 2 hours, 500 μl 1% osmium tetroxide solution (Electron Microscopy Services, Hatfield, PA) was added to each femur placed in a 1.5-mL microtube. Bones were stained in the fume hood for 5 days at room temperature with two intermediate changes of freshly made osmium tetroxide solution. Osmium solution was carefully removed and decontaminated into a small liquid waste container that had been filled with corn oil. Bones were washed under tap water for 3 h at room temperature, transferred to a new 1.5 mL microtube containing 1 mL PBS buffer and proceeded for microCT scanning.

### 2.7 Quantitative expression analysis

Total RNA was extracted from the whole femur tissue (no flushing) using a monophasic solution of guanidine isothiocyanate and phenol according to the manufacturer’s instructions (TRI Reagent, MRC, Cincinnati, OH, USA). After removal of DNA remnants with DNase I treatment (Sigma‐Aldrich, St. Louis, MO, USA), first‐strand cDNA was synthesized using 2 μg of total RNA and M‐MLV (Invitrogen). Templates were amplified with HOT FIREPol^®^ EvaGreen Master Mix (Solis Biodyne) on the CFX96 Connect real time PCR instrument (Bio-Rad Laboratories). Data analysis was performed following the 2^-ΔΔCT^ method. The primers were purchased from Eurofins Genomics and are listed in **Supplementary Table 1**. For each experiment at least three biological and two technical replicates were used.

### 2.8 Serum bone markers and free calcium

The following serum parameters were measured in stored frozen (-80°C) serum samples while samples of individual mice were assayed in the same run: total calcium and albumin (Dimension Intergrated Chemistry System Analyzer, Siemens Healthcare Diagnostics Inc., Newark (DE),U.S.A.), [Calcium intraassay coefficient of variation (CV) ≤3.0%, interassay CV ≤4.5%], [Albumin intraassay coefficient of variation (CV) ≤ 1.6%, interassay CV ≤ 2.4%]; N-terminal propeptide of type I procollagen (PINP) [Rat/Mouse PINP EIA, ELISA, Immunodiagnostics Systems Inc., Boldon, UK], [intraassay coefficient of variation (CV) ≤11.8%, interassay CV ≤11.3%]; and C-terminal telopeptides of type I collagen (CTX-I) [Rat-Laps (CTX-I) EIA, ELISA, Immunodiagnostics Systems Inc., Boldon, UK], [intraassay coefficient of variation (CV) ≤13.0%, interassay CV ≤14.0%].

### 2.9 Statistical analysis

All results are represented as box-plots showing each data point, the median and the interquartile range. Bone histomorphometry data are present as mean values ± standard deviation (SD). Statistical significance was assessed by one-way analysis of variance (ANOVA) and Tukey *post-hoc* test was performed to compare means of multiple groups. Two-way ANOVA and Tukey *post-hoc* was performed to compare means of BD values acquiring from imaging analysis among multiple groups. For datasets with small sizes (n=4-5) the assumption of normality was evaluated using the Shapiro–Wilk test and no significant deviations from normality were detected (all p>0.05). Therefore, parametric ANOVA with Tukey’s *post-hoc* test was applied to these datasets. For all tests, p<0.05 was considered statistically significant, *p< 0.05, **p< 0.01, ***p< 0.001, ****p< 0.0001.

## 3 Results

### 3.1 Modeling rebound bone loss following denosumab discontinuation in the TgRANKL osteoporosis mouse model

We investigated the development of rebound bone loss following denosumab discontinuation in the osteoporosis mouse model TgRANKL, characterized by progressive trabecular bone loss, cortical porosity and BMAT formation (17). Treatment with denosumab initiated at 6-week-old TgRANKL mice, that already developed bone loss in order to validate its therapeutic benefits. Thus, we implemented a therapeutic protocol scheme (**Figure 1A**), including experimental group I: WT-vehicle mice, II: TgRANKL-vehicle mice, III: TgRANKL mice continuously treated with denosumab for 18 weeks (TgRANKL+Dmab), IV: TgRANKL mice treated with denosumab for 6 weeks and then denosumab discontinuation for 12 weeks (TgRANKL+Dmab/Discontinuation). To track the rebound bone loss phenomenon after denosumab discontinuation, we performed *in vivo* X-ray imaging every 6 weeks in all mice for monitoring the progression of bone density in distal femurs. Treatment was well tolerated by all experimental animal groups during the intervention period. We did not observe any acute toxicities nor any significant alterations in body weights among the treated groups during the experimental study (**Supplementary Figures 1**, **2**).

X-ray *in vivo* imaging verified a 23% lower femoral bone density on average in 6-week-old TgRANKL mice compared to WT littermates in both sexes (**Figure 1B**, **Supplementary Table 2**). However, a 6 week-treatment period with denosumab was sufficient to fully rescue the osteoporotic phenotype of TgRANKL mice as shown by a 28% increase in femoral bone density, reaching the baseline levels of WT-vehicle mice (mean bone density (BD); WT: 2.11, TgRANKL-vehicle:1.52 vs TgRANKL+Dmab: 2.08, p<0.05) (**Figure 1B**, **Supplementary Table 2**). As expected based on the known denosumab pharmacokinetics, discontinuation after 6 weeks of treatment in TgRANKL mice, resulted in a continued rise in bone density for the following 6 weeks, followed by a decline to TgRANKL-vehicle levels over a subsequent 6-week period by 24 weeks of age (mean BD; female: 40%, male: 30%). However, continuous treatment of TgRANKL mice with denosumab over an 18 week-period between week 6 to 24 progressively increased bone density in both sexes. These findings indicate that discontinuation of denosumab for 12 weeks led to a pronounced osteoporotic phenotype, comparable to that observed in the TgRANKL-vehicle group, (mean BD-female; TgRANKL-vehicle: 1.21 vs TgRANKL+Dmab/Discontinuation: 1.17, p>0.05) closely mirroring the rebound effect typically observed following denosumab cessation in osteoporotic patients (**Figure 1B**).

**Figure 2:**
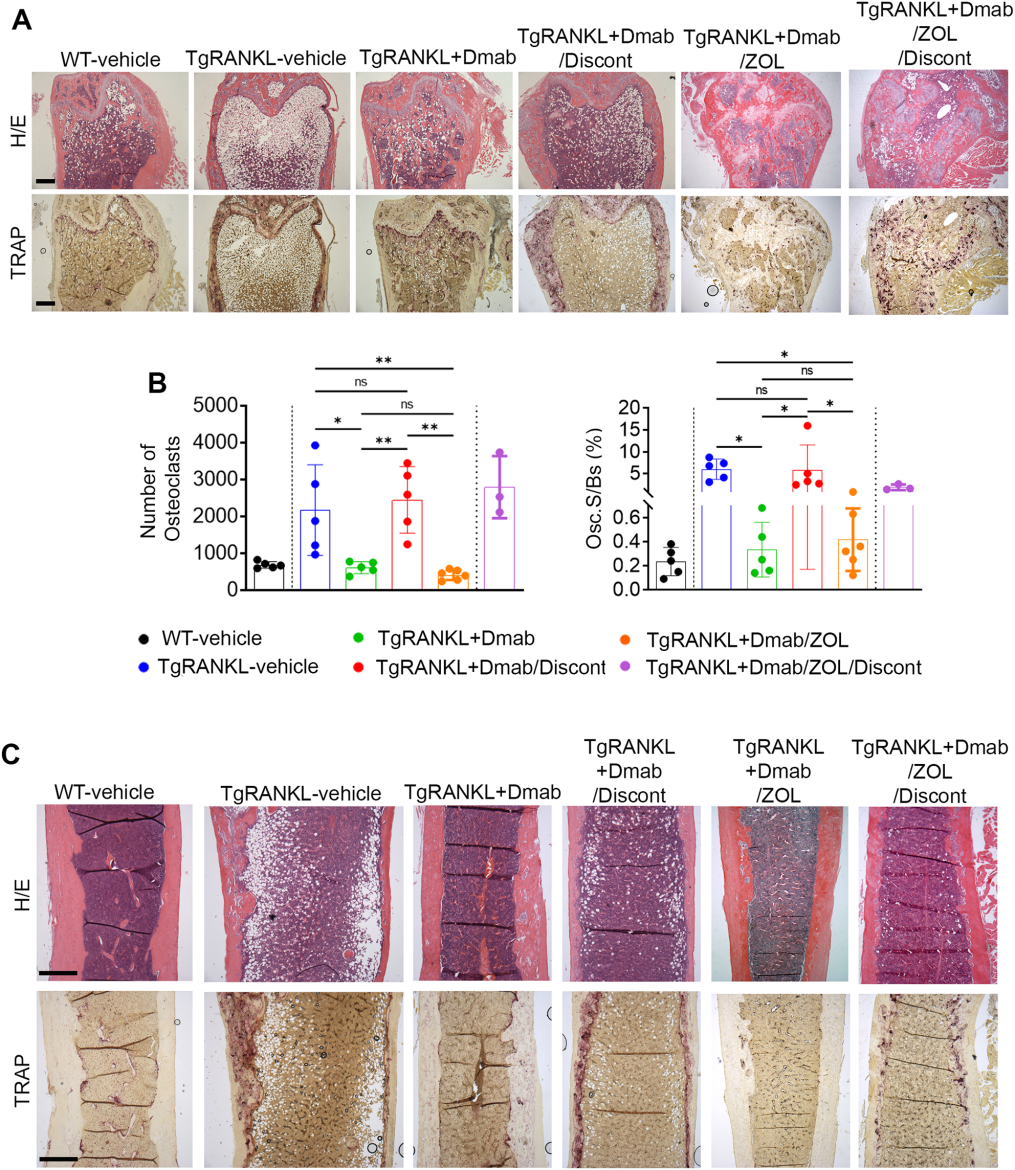
Excessive trabecular and cortical bone loss and increased osteoclast number in TgRANKL mice after denosumab discontinuation. **(A)** Representative serial sections of metaphyseal distal femurs from each group stained with hematoxylin/eosin (H/E) and TRAP (n=5 per group). Scale bar=500mm. **(B)** TRAP staining was measured as osteoclast number and osteoclast surface fraction (Oc.S/BS, %) in metaphyseal region of distal femurs. **(C)** Representative serial sections of mid‐diaphysis cortical bone stained with H/E and TRAP. Scale Bar=500mm. Data from TRAP quantification represent mean values ± SD. One-Way ANOVA and Tukey *post-hoc* test was performed. WT and TgRANKL+Dmab/ZOL/Discont mice were excluded from statistical analysis and separated from other groups using a vertical dot line in all presented graphs. ns, not statistically significant, *P<0.05, **P<0.01.

The femoral bone structure was further examined with histological analysis both at the trabecular and cortical areas. As expected, 24-week-old mice of the TgRANKL-vehicle group displayed a severely osteoporotic phenotype as shown by absence of trabecular bone, cortical porosity, increased numbers of osteoclasts and BMAT expansion, that was rescued by continuous denosumab treatment (**Figure 2**). However, denosumab discontinuation led to a rapid trabecular bone loss, cortical porosity and increased osteoclast number (**Figures 2A, B**), resulting in a phenotype reminiscent of the TgRANKL-vehicle mice, confirming the rebound effect of denosumab discontinuation (**Figure 2**).

**Figure 3:**
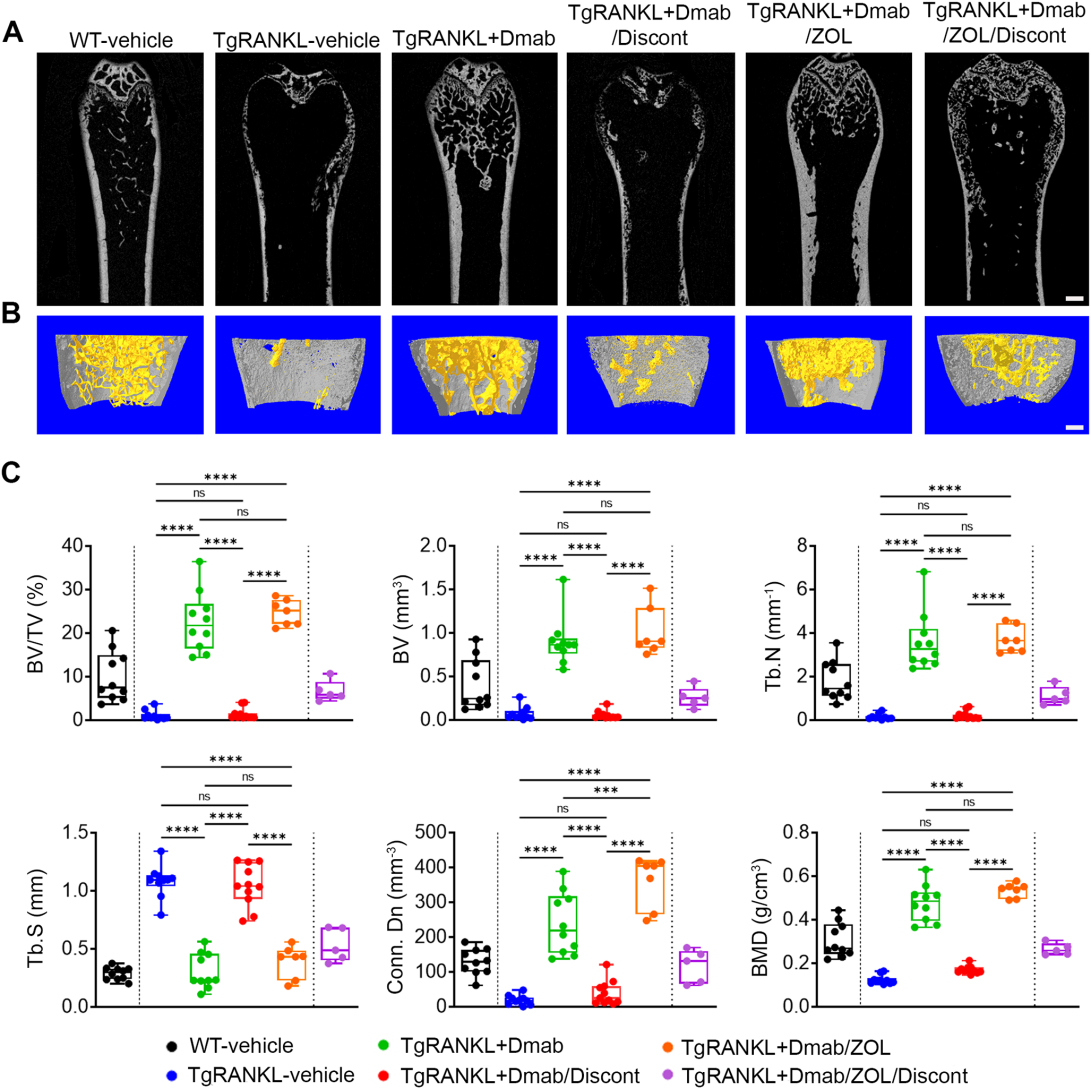
Denosumab discontinuation results in rebound trabecular bone loss in TgRANKL mice which is rescued by switch to zoledronate. **(A)** Representative 2D longitudinal and **(B)** 3D reconstructed microCT images of trabecular (yellow) and cortical bone (grey) at metaphyseal region in distal femur from each study group, scale bar=500μm. **(C)** Quantitative analysis of the trabecular bone in the metaphyseal region of the distal femurs in treated mice (n=5–11 per group) with microCT. BV/TV (Bone volume/Tissue volume, %), BV (Bone volume, mm^3^), Tb.N (Trabecular number per mm), Tb.S (Trabecular separation, mm), Conn.Dn. (Connectivity density, mm^-3^), BMD (Bone mineral density, g/cm^3^). Data are shown as median values and interquartile range. One-Way ANOVA and Tukey *post-hoc* test was performed for statistical analysis. WT and TgRANKL+Dmab/ZOL/Discont mice were excluded from statistical analysis and separated from other groups using a vertical dot line in all presented graphs. ns, not statistically significant, ***P<0.001, ****P<0.0001.

**Figure 4:**
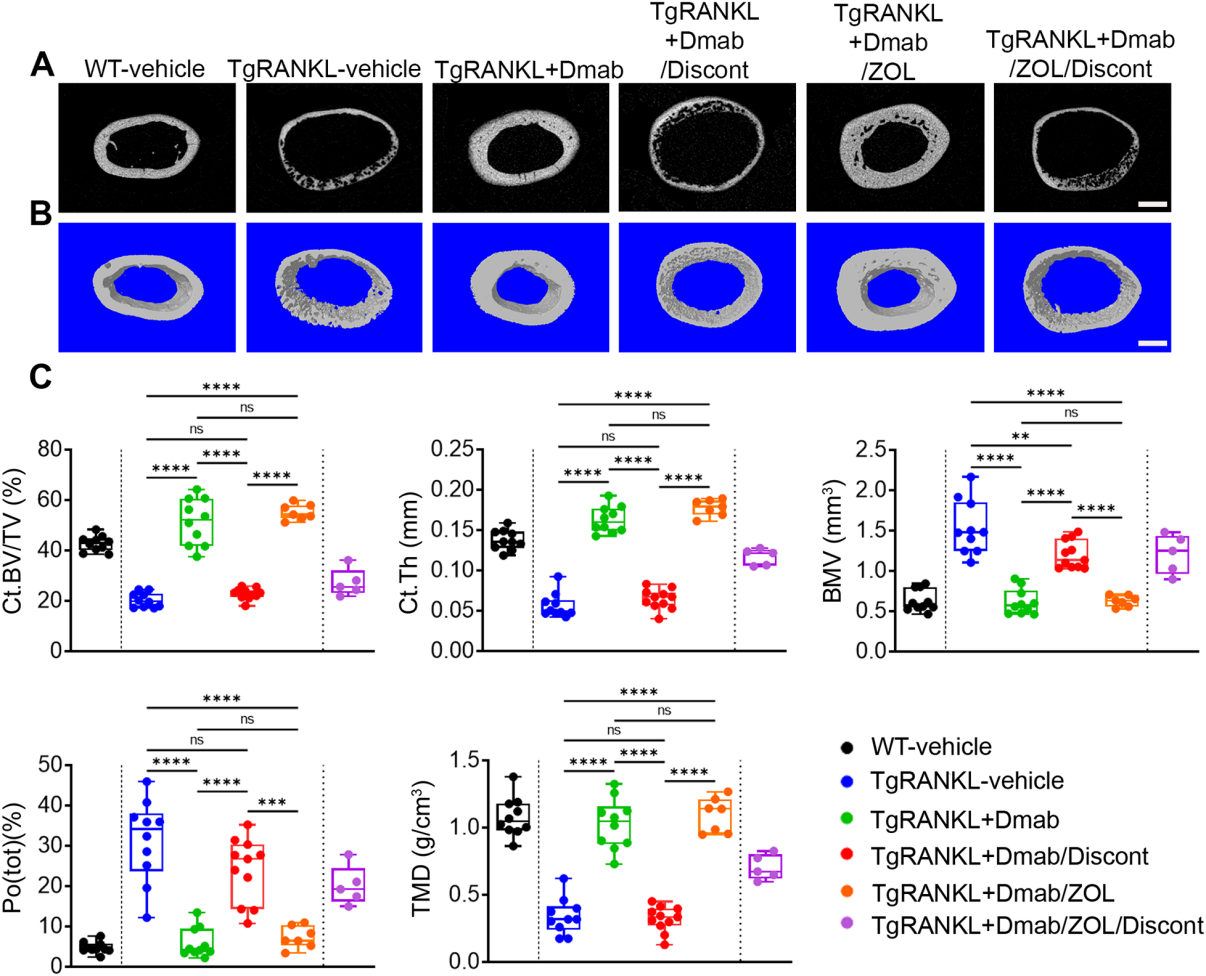
Denosumab discontinuation results in rebound cortical bone loss and porosity in TgRANKL mice which is rescued by switch to zoledronate. Representative **(A)** 2D and **(B)** 3D microCT reconstructions of cortical bone at mid-diaphysis region in distal femurs from each study group, scale bar=500μm. **(C)** Quantitative analysis of midshaft cortical bone from each group with microCT (n=5–11 per group). Ct.BV/TV (Cortical bone volume/Tissue volume, %), Ct.Th (Cortical thickness, mm), BMV (Bone marrow volume, mm^3^), Po(tot)(%) (Total porosity, %), TMD (Tissue mineral density, g/cm^3^). Data are shown as median values and interquartile range. One-Way ANOVA and Tukey *post-hoc* test was performed for statistical analysis. WT and TgRANKL+Dmab/ZOL/Discont mice were excluded from statistical analysis and separated from other groups using a vertical dot line in all presented graphs. ns: not statistically significant, **P<0.01, ***P<0.001, ****P<0.0001.

High-resolution imaging of femoral bone microarchitecture with quantitative microCT analysis confirmed that continuous denosumab treatment rescued trabecular bone loss displayed in TgRANKL-vehicle mice while denosumab discontinuation led to a rapid trabecular bone loss over 93% compared to TgRANKL+Dmab group and recurrence of the osteoporotic phenotype (**Figure 3**, **Supplementary Table 3**). Similarly, microCT analysis of cortical bone in mid-diaphysis of femurs revealed significant cortical bone loss and increased cortical porosity in TgRANKL-vehicle mice compared to WT controls, that was rescued with denosumab treatment (**Figure 4**, **Supplementary Tables 3**, **S4**). Denosumab discontinuation resulted in rebound cortical loss and porosity similarly to TgRANKL-vehicle group, as shown by the dramatic decrease of the cortical bone fraction (44%), the cortical thickness (62%) and the bone mineral density (65%) (**Figure 4C**). In addition to the femurs, rebound bone resorption was also observed in the lumbar vertebrae following denosumab discontinuation (**Supplementary Figure 3**, **Supplementary Table 5**).

**Figure 5:**
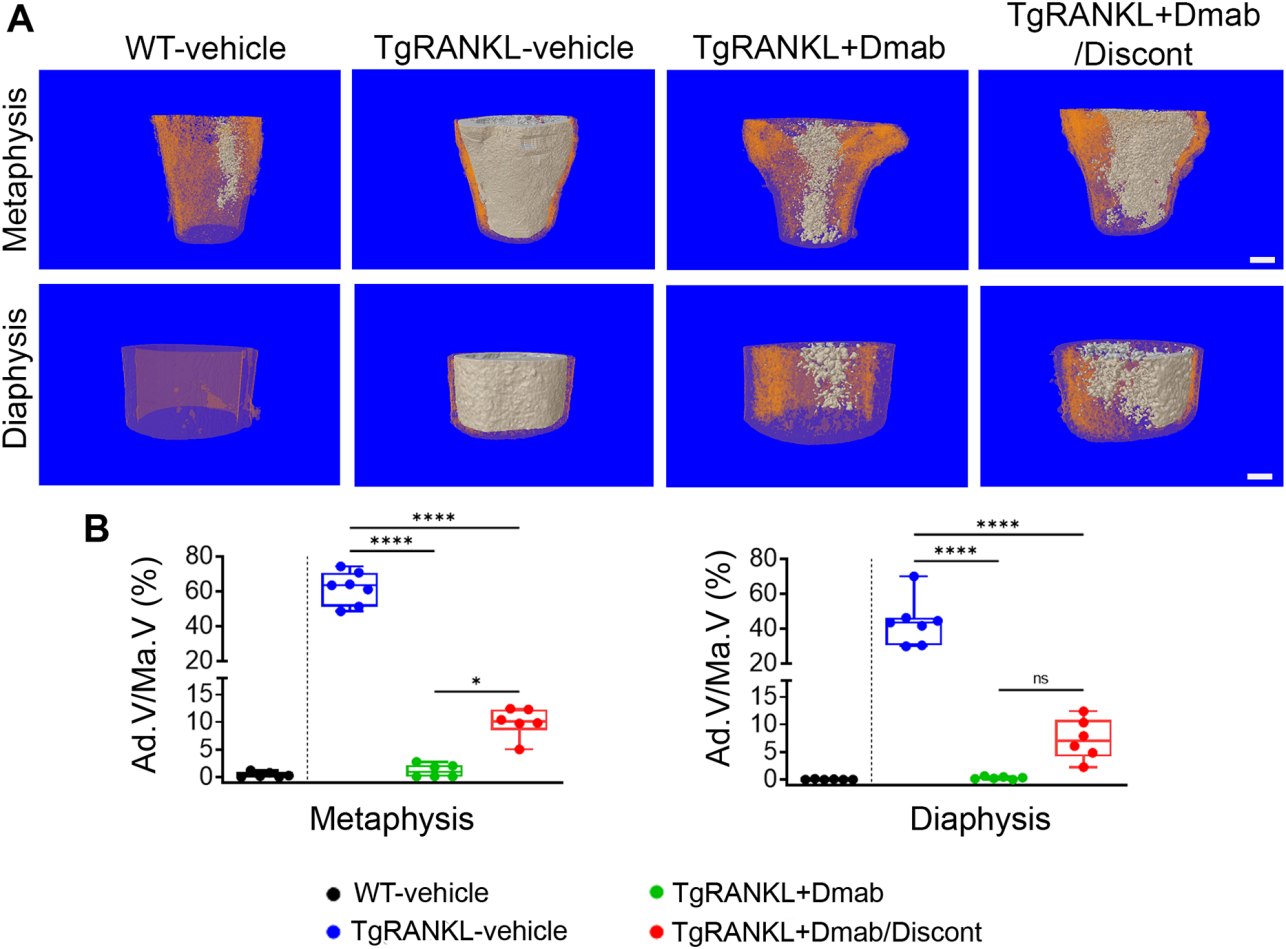
Denosumab treatment ameliorates BMA in TgRANKL mice while its discontinuation leads to a moderate reformation of BMAT. **(A)** Representative 3D reconstructed images of BMAT (white) and cortical bone (orange) at metaphyseal region and at midshaft of distal femurs from WT-vehicle, TgRANKL-vehicle, TgRANKL+Dmab and TgRANKL+Dmab/Discont groups, scale bar=500μm. **(B)** Quantitative analysis of BMAT at metaphysis and diaphysis region of distal femurs from male WT, TgRANKL-vehicle, TgRANKL+Dmab and TgRANKL+Dmab/Discont groups (n=6-7). Ad.V/Ma.V (Adipocyte Volume/Marrow Volume, %). Data are shown as median values and interquartile range. One-Way ANOVA and Tukey *post-hoc* test was performed for statistical analysis. WT and TgRANKL+Dmab/ZOL/Discont mice were excluded from statistical analysis and separated from other groups using a vertical dot line in all presented graphs. ns, not statistically significant, *P<0.05, ****P<0.0001.

Our previous work showed that TgRANKL mice of both sexes exhibit a marked expansion of BMAT compared to WT littermates, which is prevented by denosumab treatment (17). Therefore, we examined whether discontinuation of denosumab influences BMAT accumulation by performing histological analysis and microCT quantification using osmium tetroxide staining. Our analysis demonstrated that continuous denosumab treatment suppressed BMAT expansion in the distal femurs, reducing the adipocyte volume fraction by over 93% compared to the TgRANKL-vehicle group (**Figure 5**). Interestingly, the denosumab discontinuation group showed a moderate rebound in BMAT formation (11-fold on average), which was less pronounced than in the TgRANKL-vehicle group (71-fold on average) relative to the TgRANKL+Dmab group (**Figure 5**, **Supplementary Table 6**).

### 3.2 Switch to zoledronate after denosumab treatment sustains the beneficial effects of RANKL inhibition

Furthermore, we investigated the efficacy of sequential therapy with zoledronate following treatment with denosumab to sustain the anti-resorptive effects of RANKL inhibition. Thus, in the present study we included group V: TgRANKL mice treated with denosumab for 6 weeks and then switched to zoledronate treatment for 12 weeks (TgRANKL+Dmab/ZOL) (**Figure 1A**). Imaging analysis revealed a progressive increase of bone density during the treatment period at TgRANKL+Dmab/ZOL, similarly to continuous denosumab treatment (mean BD; TgRANKL+Dmab: 2.45 vs TgRANKL+Dmab/ZOL: 2.68, p>0.05) (**Figure 1B**, **Supplementary Table 2**). Histological analysis at the 24-week endpoint demonstrated that zoledronate treatment following denosumab therapy effectively sustained the protective effects of denosumab, by reducing osteoclast numbers and preserving both trabecular and cortical bone structures (**Figure 2**). MicroCT analysis confirmed that switching to zoledronate treatment sustained the effects of denosumab in the trabecular bone leading to higher bone volume fraction and BMD compared to WT, TgRANKL and TgRANKL+Dmab/Discont group, displaying similar values with TgRANKL+Dmab group mice (BV/TV (%); TgRANKL+Dmab: 22.63, TgRANKL+Dmab/ZOL: 24.72, p>0.05) (**Figure 3**, **Supplementary Table 3**). Moreover, sequential zoledronate treatment fully rescued the cortical bone structure displaying a similar bone profile with the continuous denosumab treatment as shown by 3D microCT images and cortical bone parameters (**Figure 4**, **Supplementary Tables 3**, **S4**).

In our experimental design, all mice were sacrificed at week 24, except for a small subset from the Dmab/ZOL group (n=5), which were retained to monitor bone turnover following zoledronate discontinuation (TgRANKL+Dmab/ZOL/Discont group). Imaging showed that mice in the zoledronate discontinuation group preserved the peak bone density attained with Dmab/ZOL treatment for a duration of 12 weeks, while a sharp decline in bone density occurred over a 6-week period at 42 weeks of age (BD reduction; female: 13%, male: 29%) (**Figure 1B**, **Supplementary Table 2**). Histological evaluation of TgRANKL+Dmab/ZOL/Discont mice at week 42 demonstrated a pronounced loss of trabecular bone, increased cortical porosity, and elevated numbers of osteoclasts (**Figures 2A–C**). MicroCT analysis confirmed the bone loss developed in the TgRANKL+Dmab/ZOL/Discont group (**Figures 3A–C**).

### 3.3 Bone turnover biomarkers in denosumab discontinuation and sequential zoledronate

**Figure 6:**
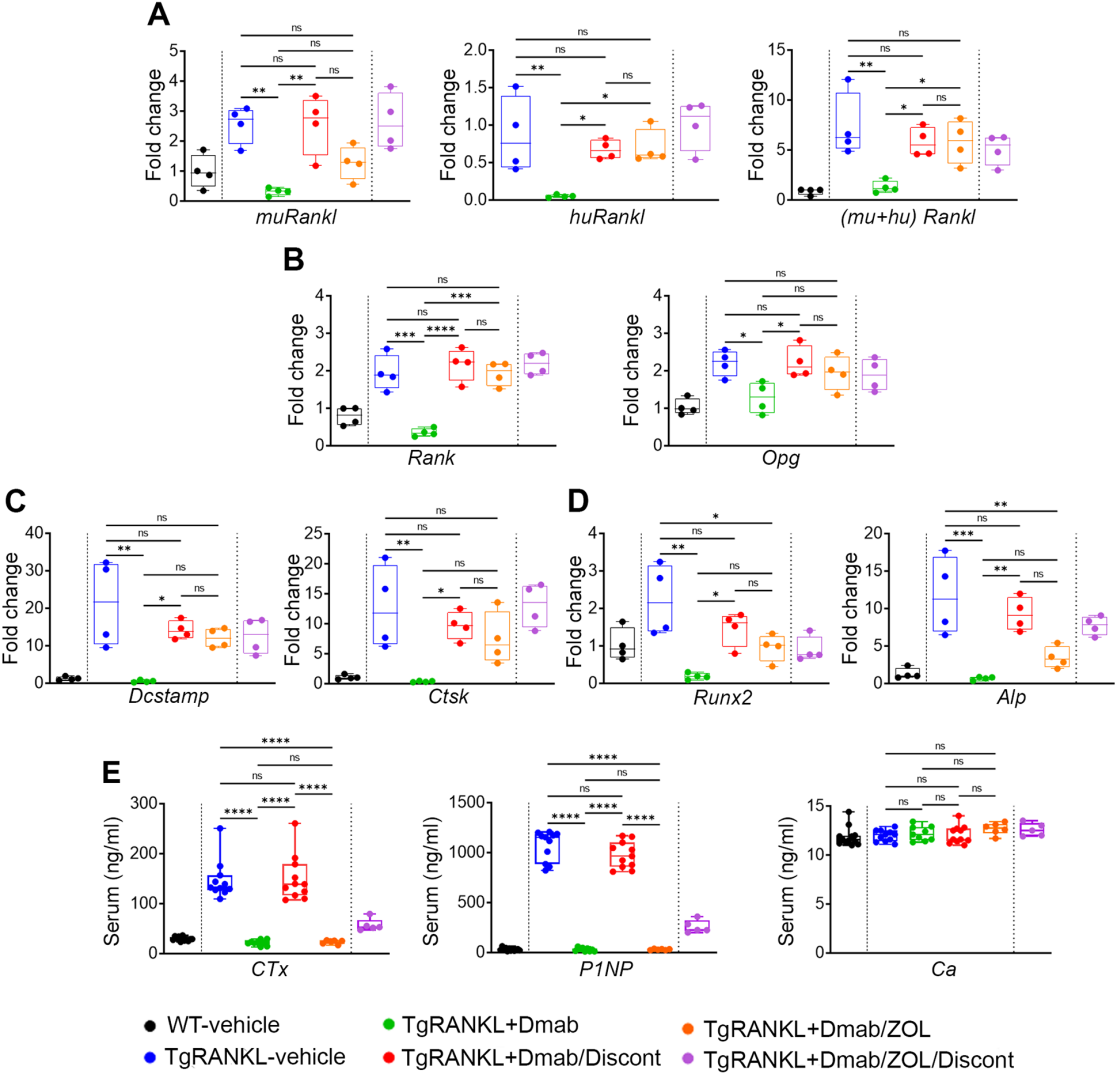
Comparative gene expression analysis and estimation of bone turnover markers in blood serum. Comparative mRNA expression analysis for **(A)** mouse, human and total (mouse+human) *Rankl* genes, **(B)** *Rank* and *Opg*, **(C)** *Dcstamp*, and *Ctsk* for osteoclast activity and **(D)** *Runx2*, and *Alp* for osteoblast activity in femurs among experimental groups (n=4 per group). Relative fold change of mRNA expression is normalized against *GAPDH*. **(E)** Serum levels of Ctx, P1NP and free calcium (Ca) among tested groups (n=5–11 per group). Gene expression and serum data are shown as median values and interquartile range. One-Way ANOVA and Tukey *post-hoc* test was performed for statistical analysis. WT and TgRANKL+Dmab/ZOL/Discont mice were excluded from statistical analysis and separated from other groups using a vertical dot line in all presented graphs. ns, not statistically significant, *P<0.05, **P<0.01, ***P<0.001, ****P<0.0001.

The gene expression profile regulating bone remodeling was analyzed in femurs from all experimental groups, with or without therapeutic intervention, using qPCR. This included assessment of RANKL/RANK/OPG signaling components, osteoclast markers (*Dcstamp* and *Ctsk*), and osteoblast markers (*Runx2* and *Alp*). Furthermore, blood serum levels of bone turnover markers (BTMs), CTx and P1NP as well as calcium were measured at the endpoint (**Figure 6**).

Continuous denosumab treatment had the most dramatic reduction in all above markers compared to TgRANKL-vehicle group, indicating profound suppression of bone turnover to baseline levels. Discontinuation led to the loss of denosumab’s beneficial effects, as indicated by bone turnover markers rising to levels observed in the TgRANKL-vehicle group. Switch to zoledronate led to a moderate reduction in the expression of genes associated with bone remodeling compared to the TgRANKL-vehicle group, while serum bone markers decreased to levels comparable to those observed in the TgRANKL+Dmab group. Zoledronate discontinuation produced gene expression patterns similar to those seen in the TgRANKL/Dmab/Zol group (**Figures 6A–D**). However, serum levels of CTx and P1NP were elevated compared to this group, indicating increased bone turnover. At last, the circulating levels of free calcium in blood serum remained unchanged among experimental intervention schemes and vehicle treated groups, respectively (**Figure 6E**).

## 4 Discussion

Discontinuation of denosumab poses a critical challenge due to a pronounced rebound phenomenon, characterized by rapid BMD loss and significant increases in BTMs (e.g., CTx and P1NP), often surpassing pretreatment levels (7, 23). Even brief treatment discontinuations can lead to rapid reversal of therapeutic gains, highlighting the clinical importance of effectively managing rebound bone loss (8, 24). However, preclinical studies examining the rebound effect after denosumab discontinuation have been limited, primarily because denosumab is not cross-reactive with murine RANKL (12). Discontinuation of a single-injection anti-mouse RANKL treatment in OVX mice, failed to replicate the strong clinical rebound bone loss seen in patients, showing only a modest response without significance (25). More recently, additional studies successfully induced a rebound effect using either osteoprotegerin-Fc (OPG-Fc) discontinuation in WT mice (26, 27), anti-RANKL antibody discontinuation in healthy or ovariectomized mice allowing investigation of discontinuation-induced overshoot (16) or denosumab discontinuation in a murine model introducing the human epitope into the endogenous *Rankl* gene (15). While these models provide valuable mechanistic insights, their clinical relevance is limited by their reliance on non-osteoporotic baseline conditions in healthy animal models, use of surrogate RANKL inhibitors (OPG-Fc and non-humanized antibodies) rather than denosumab, and the shorter pharmacokinetics and reduced anti-resorptive potency of OPG-Fc compared to denosumab (28). To address these limitations, we utilized TgRANKL mice, a transgenic model expressing huRANKL that develops an osteoporotic phenotype with enhanced osteoclastogenesis, trabecular bone loss, cortical porosity, and elevated bone turnover in both sexes, features that are effectively reversed by denosumab treatment (17). In this study, we investigated rebound bone loss following denosumab discontinuation in the osteoporotic TgRANKL mouse model and assessed the efficacy of subsequent zoledronate treatment.

Treatment of 6-week-old TgRANKL mice with denosumab for 6 weeks rescued the osteoporotic phenotype and increased the bone density compared to the TgRANKL-vehicle group. Extending denosumab therapy for additional 12 weeks led to further bone density gains. However, upon discontinuation after 6 weeks of denosumab treatment, a pronounced rebound effect was observed, though not immediately. Bone density continued to rise during the first 6 weeks after stopping treatment (weeks 12 to 18), followed by a rapid decline in the subsequent 6 weeks (weeks 18 to 24) in both sexes. This timeline matched rebound observations following OPG-Fc discontinuation in WT mice (13). These results suggest a general tendency of the skeleton to revert to disease baseline conditions upon cessation of potent antiresorptive therapy, reflecting the concept of mechanotactic reset to lower bone mass (29). To further elucidate these findings, we conducted detailed histological and microCT analyses. Histological evaluation revealed significant trabecular and cortical bone loss post-discontinuation, accompanied by increased osteoclast numbers, consistent with human biopsy findings post-denosumab cessation (30). MicroCT demonstrated severe trabecular and cortical bone loss along with increased cortical porosity, indicating comparable severity between the discontinuation group and the TgRANKL-vehicle osteoporotic group. TgRANKL osteoporotic mice are characterized by a significantly high bone turnover rate as shown by the measured BTMs and gene expression analysis. In contrast, denosumab treatment significantly reduced the circulating BTMs, reflecting effective inhibition of bone remodeling. Consistent with clinical data (8), the circulating BTMs in TgRANKL mice after discontinuation of denosumab therapy rose to levels comparable to those observed in TgRANKL-vehicle mice, indicating increased bone turnover. Similarly, denosumab administration led to a reduction in the expression of osteoclastogenic genes (*Ctsk*, *Dcstamp*) within bone tissue, whereas a marked upregulation of these genes was observed in both the TgRANKL-vehicle group and following denosumab discontinuation. These findings reinforce increased osteoclast differentiation and activation as central mechanisms driving rebound bone loss. Concurrently, osteogenic markers (*Runx2*, *Alp*) also rebounded to osteoporotic levels, indicating increased bone remodeling and coupling mechanisms post-discontinuation (31). Interestingly, treatment of TgRANKL mice with denosumab resulted in reduced *Opg* expression levels, in accordance to a recent study using a denosumab-specific discontinuation knock-in mouse model (15). According to this study, denosumab treatment reduces osteoblast and osteocyte populations, resulting in diminished local OPG levels. Following denosumab cessation, reduction of OPG protein in the bone microenvironment potentially enhances RANKL-driven osteoclast differentiation and activity, thereby intensifying rebound resorption (15). These findings align with a spatial transcriptomic study (27), who demonstrated that OPG-Fc treatment leads to the accumulation of RANKL-expressing osteoprogenitors and reduced *Opg* expression in osteocytes, creating local activation sites that drive rebound resorption upon withdrawal.

BMAT is considered as an endocrine organ that can regulate bone metabolism through secretion of various paracrine factors, namely, adipokines. Although numerous studies have shown the positive effects of various antiresorptive therapies on BMAT expansion (32), no data are available regarding the effects of denosumab therapy or its discontinuation. In this study we examined how BMAT is affected upon denosumab treatment and discontinuation in TgRANKL mice that exhibit extensive BMAT expansion, inversely correlating with bone mass (21). Denosumab treatment fully suppressed BMAT progression, whereas its discontinuation led to moderate reformation of BMAT. This observation represents a novel finding that warrants further investigation. Moderate marrow adiposity reformation following denosumab discontinuation indicates that BMAT is likely a consequence of bone loss rather than the primary driver of the rebound effect, consistent with our prior work in untreated TgRANKL mice demonstrating that bone resorption precedes and drives BMAT accumulation (21). However, the contribution of BMAT to osteoclastogenesis and rebound bone resorption should not be overlooked. It is possible that BMAT reformation following denosumab discontinuation may amplify the rebound osteoclastic response through paracrine adipokine secretion. Bone marrow adipocytes produce osteoclastogenic factors including RANKL, TNF-α, IL-6, and IL-1β that enhance RANKL-mediated osteoclast precursor differentiation and activation. These factors promote a permissive microenvironment that synergizes with the increased RANKL availability observed after the cessation of RANKL inhibition (33). BMAT reformation may partially contribute to rebound *Rankl* expression shown in this study, amplifying the osteoclastogenic response as indicated by our prior fractionation studies (21). Resolving these mechanisms necessitates future investigations into BMAT-bone resorption interactions after denosumab cessation.

For patients discontinuing denosumab treatment, the recommended practice is to promptly administer a follow-up antiresorptive therapy to effectively mitigate rebound bone loss and BMD reduction, thus preserving a low risk of fractures (34). Administration of either oral bisphosphonate or intravenous zoledronate infusions in such patients is the most widely studied therapeutic scheme in randomized controlled trials and observational studies (10, 35, 36). However, the efficacy of post denosumab treatment with bisphosphonates is controversial since some studies showed no alterations of BMD values after 1 year of a single dose of zoledronate infusion (10, 35, 37) while other studies demonstrate partial protective effects (38–40). Due to these contradictions, numerous ongoing clinical studies with different designs and approaches are exploring the efficacy of zoledronate for mitigating the effects of denosumab discontinuation (41, 42). A key aspect of our study was to evaluate the effect of zoledronate as a sequential treatment following denosumab discontinuation in our preclinical model. Our results demonstrated that immediate switch to zoledronate before BMD loss occurred, effectively prevented rebound bone loss, maintaining significant improvements in BMD and bone microarchitecture achieved by denosumab treatment. These findings align with recent preclinical data demonstrating that early initiation and repeated dosing of zoledronate effectively mitigated rebound bone loss post-OPG-Fc withdrawal, underscoring the clinical relevance of our preclinical findings (26). Furthermore, we investigated whether rebound bone loss develops after zoledronate discontinuation. The Dmab/ZOL discontinuation group maintained the peak bone density achieved through Dmab/ZOL treatment for a 12-week period; however, a pronounced decline in bone density was observed over a subsequent 6-week interval. Histological and microCT analyses confirmed the occurrence of bone loss in the TgRANKL+Dmab/ZOL/Discont group. Interestingly, discontinuation of zoledronate treatment revealed a notably delayed onset of rebound compared to denosumab cessation. Stopping denosumab treatment alone resulted in severe rebound bone loss within 12 weeks, whereas discontinuation in mice treated with denosumab followed by sequential zoledronate caused bone loss after 18 weeks. This delay likely reflects zoledronate’s pharmacokinetics, characterized by strong binding to bone hydroxyapatite, resulting in prolonged antiresorptive activity after discontinuation (24, 43). Such findings suggest zoledronate may offer extended protective windows against rebound bone loss compared to therapies with rapid clearance like denosumab. Nonetheless, considerable inter-individual variation exists in the timing of bone loss following zoledronate effect depletion, with this temporal variability contingent upon antecedent denosumab treatment duration, baseline BMD values, and patient-specific determinants (44, 45). In this study, the TgRANKL mouse model of osteoporosis expressing human RANKL was employed to directly assess the skeletal outcomes of denosumab discontinuation and to examine the effectiveness of sequential zoledronate therapy. Our results demonstrate that TgRANKL mice recapitulate the clinical rebound effects observed after denosumab withdrawal. Administration of zoledronate after denosumab treatment effectively prevented rebound bone loss, highlighting its promise as a transition strategy. Our findings establish TgRANKL mice as a unique osteoporotic model for elucidating the mechanisms underlying denosumab rebound and for evaluating sequential antiresorptive treatment approaches.

## Data Availability

The raw data supporting the conclusions of this article will be made available by the authors, without undue reservation.
