# Supplementary material for: Modeling rebound bone loss following denosumab discontinuation and sequential zoledronate therapy in TgRANKL osteoporotic mice
Source: Front Endocrinol (Lausanne). 2026 Feb 26;17:1783656. doi: 10.3389/fendo.2026.1783656 (PMC12979105; doi:10.3389/fendo.2026.1783656)
Supplement: Supplementary file 1 [file DataSheet1.docx]

**
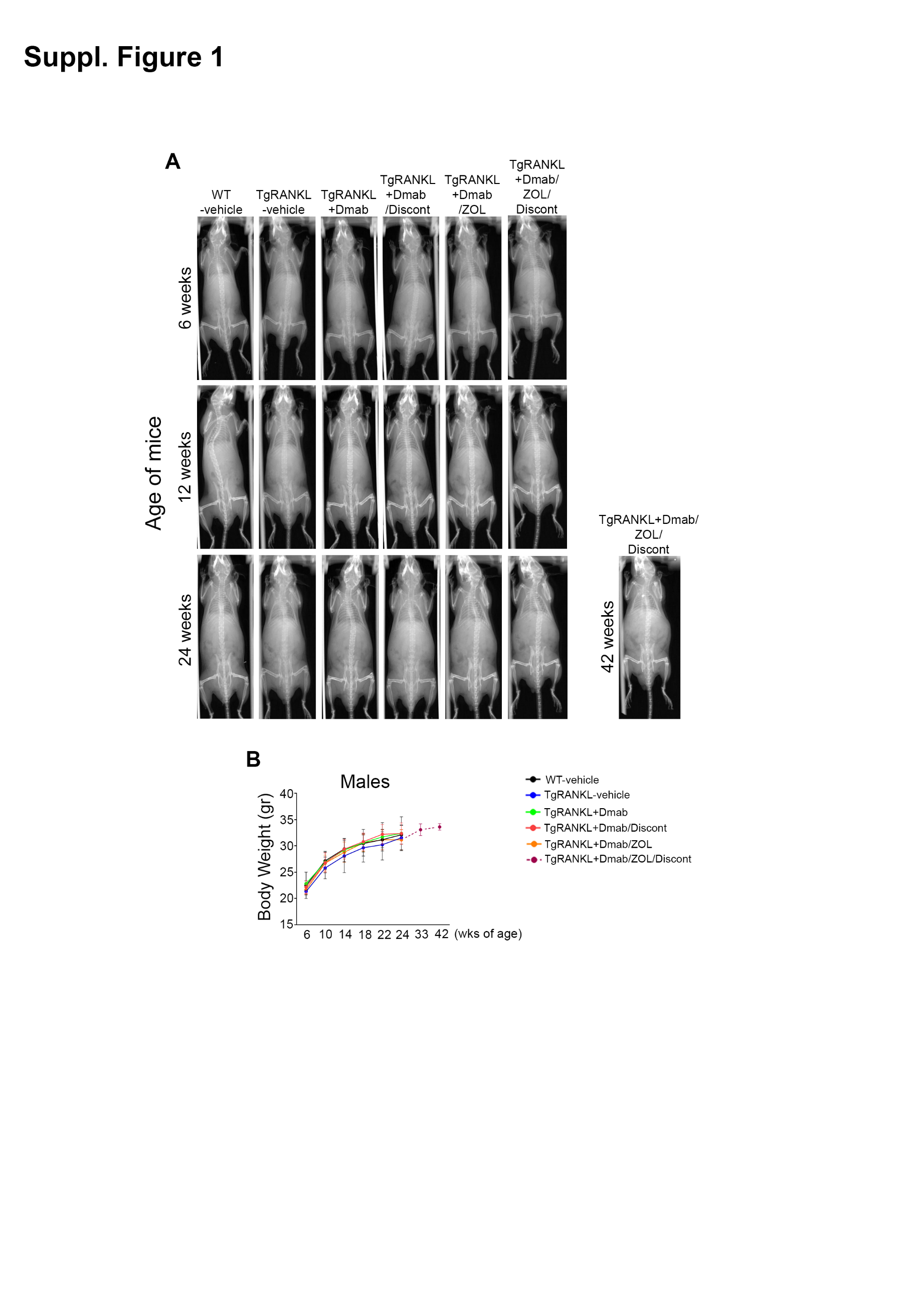
**

**Fig. S1. *In vivo* x-ray imaging in males.** (**A**) Representative conventional x-ray images of male mice from each experimental group at indicated time points using the *In-Vivo* Xtreme Imaging System (Brucker). (**B**) Body weight curve for male mice from each group during treatment period.


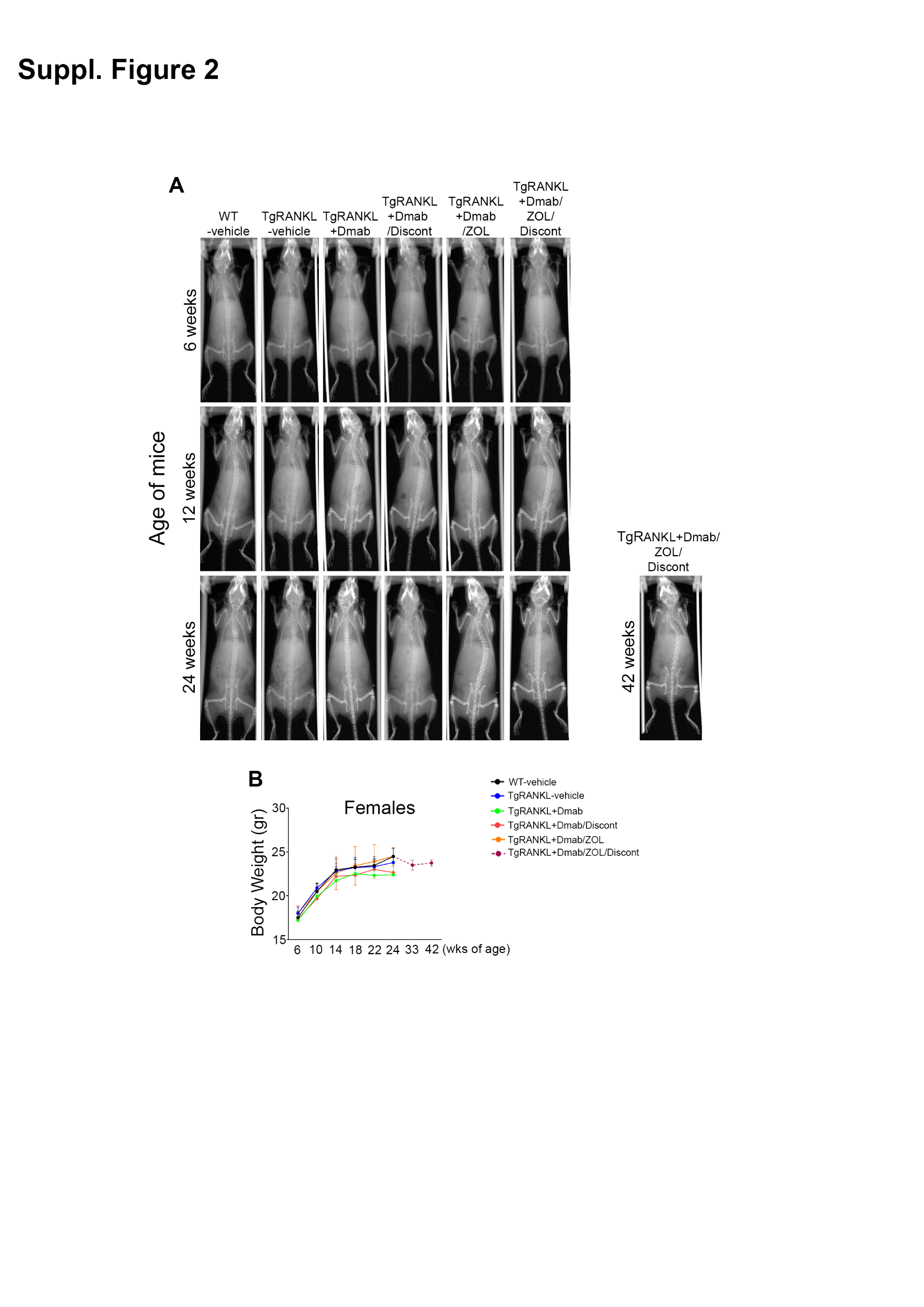


**Fig. S2. *In vivo* x-ray imaging in females.** (**A**) Representative conventional x-ray images of female mice from each experimental group at indicated time points using the In-Vivo Xtreme Imaging System (Brucker). (**B**) Body weight curve for female mice from each group during treatment period.


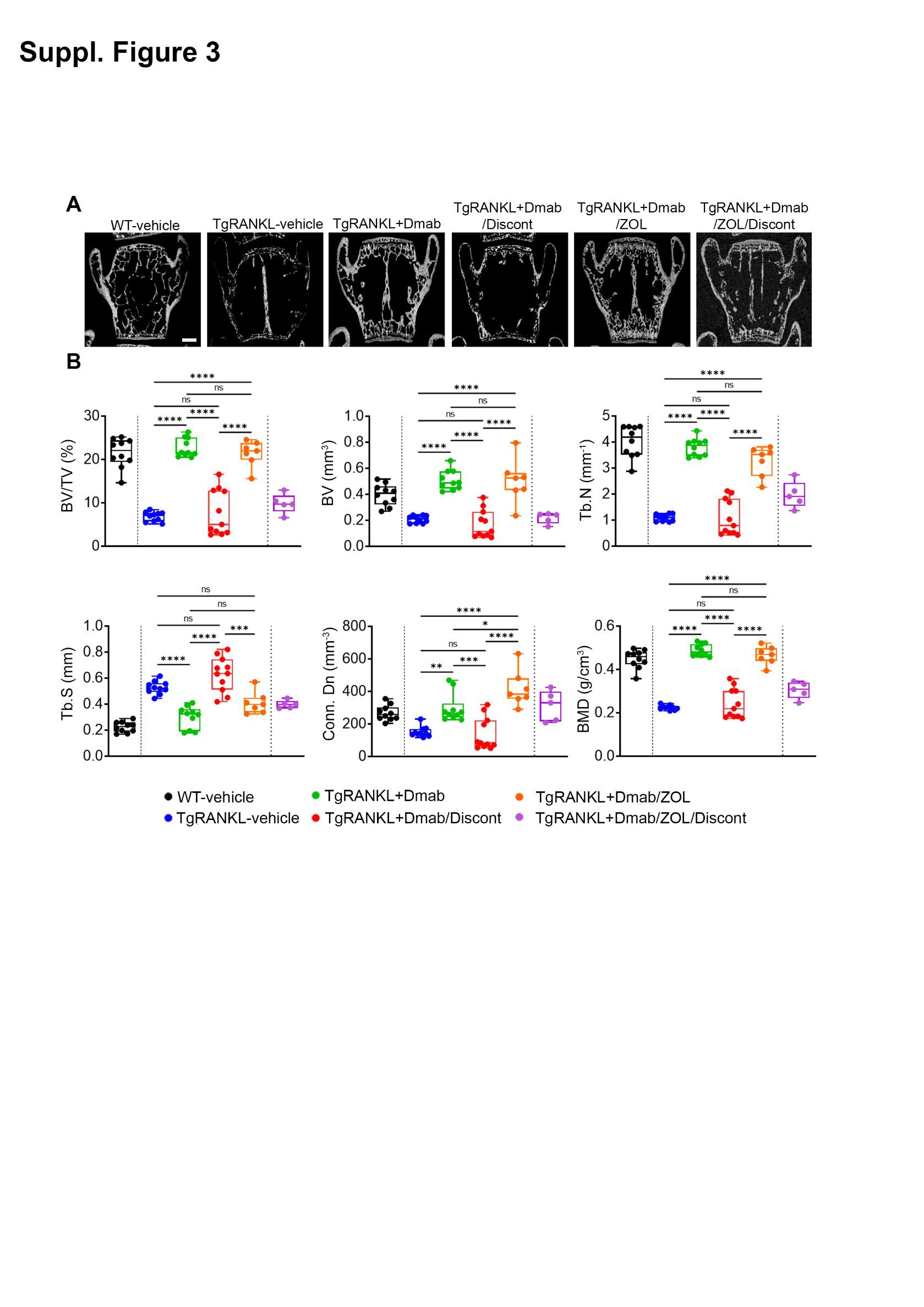


**Fig. S3. A pronounced rebound effect, after Denosumab discontinuation, is prominent in the lumbar spine in TgRANKL mice**. (**A**) Representative 2D longitudinal microCT images of lumbar spine in each group, scale bar=500μm. (**B**) Quantitative analysis of trabecular bone parameters in lumbar spine from each group (n=5-11 per group) with microCT. BV/TV (Bone volume/Tissue volume, %), BV (Bone volume, mm^3^), Tb.N (Trabecular number per mm), Tb.S (Trabecular separation, mm), Conn.Dn. (Connectivity density, mm^-3^), BMD (Bone mineral density, g/cm^3^). Data are shown as median values and interquartile range. One-Way ANOVA and Tukey post-hoc test was performed for statistical analysis. WT and TgRANKL+Dmab/ZOL/Discont mice were excluded from statistical analysis and separated from other groups using a vertical dot line in all presented graphs. ns: not statistically significant, *P<0.05, **P<0.01, ***P<0.001, ****P<0.0001.

**Table S1.** Primer sequences used for qPCR.

| **Target genes** | **Forward (5'-3')** | **Reverse (5'-3')** |
| --- | --- | --- |
| *Gapdh* | ACATCAAGAAGGTGGTGAAGCAGG | AGTTGCTGTTGAAGTCGCAGGAGA |
| (human+mouse) *Rankl* | ACCTGTACGCCAACATTTGC | CTTGGG ATTTTGATGCTGGT |
| human *Rankl* | ACGCGTATTTACAGCCAGTG | 5CCCGTAATTGCTCCAATCTG |
| mouse *Rankl* | TGTACTTTCGAGCGCAGATG | AGGCTTGTTTCATCCTCCTG |
| mouse *Opg* | TGATGTATGCCCTCAAGCAC | TTGTGAAGCTGTGCAGGAAC |
| mouse *Rank* | TCTTATGTTGGGGTCCATCC | AATAAGCTTAGCCCCGAACC |
| mouse *Dcstamp* | TGAAAAGGAGAGGCACCAAC | AGCCCAAGGGTTTTCTTCTC |
| mouse *Ctsk* | GGCTCGGAATAAGAACAACG | GCACCATGAAGGAAGGAATC |
| mouse *Runx2* | CACTGCCACCTCTGACTTCT | GCTCTCAGTGAGGGATGAAA |
| mouse *Alp* | GCAGGTTTCTCTCTTGG | CTGGGAGTCTCATCCTGAGC |

**Table S2.** Bone density measurements of distal femurs were obtained from in vivo X-ray imaging for the experimental groups throughout the study protocol. BD: Bone Density (g/cm^3^). Statistical analysis using two-way ANOVA followed by Tukey’s post-hoc test was performed exclusively on data from TgRANKL groups collected between weeks 6 and 24 of age. Data represents mean values ± SD. ^a^p<0.05 vs TgRANKL-vehicle, ^b^p<0.05 vs TgRANKL+Dmab, ^c^p<0.05 vs TgRANKL+Dmab/Discont, ^d^p<0.05 vs TgRANKL+Dmab/ZOL). BD values with red color highlights Dmab discontinuation period while with blue color ZOL discontinuation.

| **Gender** | **Age (weeks)** | **WT-vehicle** | **TgRANKL-vehicle** | **TgRANKL**  **+Dmab** | **TgRANKL**  **+Dmab/**  **Discont** | **TgRANKL**  **+Dmab/ZOL** |
| --- | --- | --- | --- | --- | --- | --- |
| Male | 6 | 1.88±0.26 | 1.38±0.29 | 1.40±0.34 | 1.30±0.30 | 1.22±0.25 |
|  | 12 | 2.70±0.72 | 1.78±0.37^b,c,d^ | 2.44±0.4^a^ | 2.77±0.55^a^ | 2.36±0.31^a^ |
|  | 18 | 2.56±0.59 | 1.65±0.33^b,c,d^ | 2.61±0.41^a^ | 3.08±0.50^a^ | 2.59±0.40^a^ |
|  | 24 | 2.77±0.40 | 1.68±0.27^b,c,d^ | 2.93±0.32^a^ | 2.17±0.23^b,d^ | 3.25±0.49^a,c^ |
|  | 30 | − | − | − | − | 3.305±0.34 |
|  | 36 | − | − | − | − | 2.944±0.44 |
|  | 42 | − | − | − | − | 2.096±0.32 |
| Female | 6 | 1.21±0.16 | 1.01±0.07 | 0.91±0.16 | 0.94±0.11 | 0.86±0.12 |
|  | 12 | 1.52±0.18 | 1.27±0.12^b,c,d^ | 1.53±0.10^a^ | 1.80±0.17^a^ | 1.63±0.23^a^ |
|  | 18 | 1.79±0.3 | 1.28±0.07^b,c,d^ | 1,75±0.24^a^ | 1.95±0.23^a^ | 1.80±0.11^a^ |
|  | 24 | 1.89±0.41 | 1.21±0.10 ^b,d^ | 1.98±0.26^a,c^ | 1.17±0.06 ^b,d^ | 2.12±0.21 ^a,c^ |
|  | 30 | − | − | − | − | 1.973±0.055 |
|  | 36 | − | − | − | − | 1.935±0.22 |
|  | 42 | − | − | − | − | 1.689±0.25 |

**Table S3**. Measurement of bone parameters through μCT analysis in metaphysis region of distal femurs among study groups. One-way analysis of variance (ANOVA) and Tukey post-hoc test was performed among TgRANKL groups (^a^p<0.05 vs TgRANKL-vehicle, ^b^p<0.05 vs TgRANKL+Dmab, ^c^p<0.05 vs TgRANKL+Dmab/Discont, ^d^p<0.05 vs TgRANKL+Dmab/ZOL). Student’s t-test was performed between WT and TgRANKL vehicle groups (*<0.05). Data are presented as mean values ± SD. Trabecular bone structure was assessed with BV/TV, bone volume/tissue volume; Tb.Sp, trabecular separation; Tb.N, trabecular number; Conn.Dn, connectivity density; BMD, bone mineral density. Cortical bone structure was assessed with Ct.BV/TV, cortical bone volume/tissue volume; BMV, bone marrow volume; Ct.Th, cortical thickness; Po(tot), total cortical porosity; TMD, tissue mineral density.

| **Trabecular Bone Parameters** | **WT-vehicle** | **TgRANKL-vehicle** | **TgRANKL**  **+Dmab** | **TgRANKL**  **+Dmab**  **/Discont** | **TgRANKL**  **+Dmab/ZOL** | **TgRANKL**  **+Dmab/ZOL/**  **Discont** |
| --- | --- | --- | --- | --- | --- | --- |
| BV/TV (%) | 10.00 ± 5.78 | 1.1 ± 1.13^*,b,d^ | 22.63 ± 6.8 ^a,c^ | 1.41 ± 1.32 ^b,d^ | 24.72 ± 2.96 ^a,c^ | 6.69 ± 2.41 |
| BV (mm) | 0.40 ± 0.29 | 0.07 ± 0.07^*,b,d^ | 0.90 ± 0.27 ^a,c^ | 0.05 ± 0.04 ^b,d^ | 1.00 ± 0.27 ^a,c^ | 0.25 ± 0.11 |
| Tb.Sp (mm) | 0.28 ± 0.05 | 1.08 ± 0.13^*,b,d^ | 0.30 ± 0.15 ^a,c^ | 1.04 ± 0.18 ^b,d^ | 0.39 ± 0.13 ^a,c^ | 0.53 ± 0.14 |
| Tb.N (mm^-1^) | 1.81 ± 0.9 | 0.15 ± 0.13^*,b,d^ | 3.60 ± 1.33 ^a,c^ | 0.21 ± 0.19 ^b,d^ | 3.68 ± 0.61 ^a,c^ | 1.11 ± 0.42 |
| Conn.Dn ( mm^-3^) | 129.5 ± 37.67 | 19.26 ± 13.95^*,b,d^ | 238.9 ± 89.21 ^a,c,d^ | 39.55 ± 34.96 ^b,d^ | 360.7 ± 73.11 ^a,c^ | 116.9 ± 48.05 |
| BMD (g/cm^3^) | 0.30± 0.08 | 0.12 ± 0.02^*,b,d^ | 0.48 ± 0.08 ^a,c^ | 0.17 ± 0.01 ^b,d^ | 0.53 ± 0.03 ^a,c^ | 0.26 ± 0.02 |
| **Cortical Bone Parameters** | **WT-vehicle** | **TgRANKL-vehicle** | **TgRANKL**  **+Dmab** | **TgRANKL**  **+Dmab**  **/Discont** | **TgRANKL**  **+Dmab/ZOL** | **TgRANKL**  **+Dmab/ZOL/**  **Discont** |
| Ct.BV/TV (%) | 42.74 ± 3.09 | 20.33 ± 2.92^*,b,d^ | 51.37 ± 9.22 ^a,c^ | 22.82 ± 2.21 ^b,d^ | 55.27 ± 3.10 ^a,c^ | 27.17 ± 5.49 |
| BMV (mm^3^) | 0.63 ± 0.13 | 1.53 ± 0.33^*,b,c,d^ | 0.61 ± 0.15 ^a,c^ | 1.20 ± 0.17 ^a,b,d^ | 0.62 ± 0.06 ^a,c^ | 1.20 ± 0.24 |
| Ct.Th (mm) | 0.13 ± 0.01 | 0.05 ± 0.01^*,b,d^ | 0.16 ± 0.01 ^a,c^ | 0.06 ± 0.01 ^b,d^ | 0.17 ± 0.01 ^a,c^ | 0.11 ± 0.01 |
| Po(tot) (%) | 4.75 ± 1.40 | 30.86 ± 9.77^*,b,d^ | 5.92 ± 3.65 ^a,c^ | 24.03 ± 7.92 ^b,d^ | 7.31 ± 2.73 ^a,c^ | 20.12 ± 4.87 |
| TMD (g/cm^3^) | 1.07 ± 0.14 | 0.34 ± 0.13^*,b,d^ | 1.02 ± 0.18 ^a,c^ | 0.32 ± 0.09 ^b,d^ | 1.09 ± 0.12 ^a,c^ | 0.70 ± 0.09 |

**Table S4**. Measurement of bone parameters through μCT analysis in metaphysis region of distal femurs among study groups for each sex. One-way analysis of variance (ANOVA) and Tukey post-hoc test was performed among TgRANKL groups (^a^p<0.05 vs TgRANKL-vehicle, ^b^p<0.05 vs TgRANKL+Dmab, ^c^p<0.05 vs TgRANKL+Dmab/Discont, ^d^p<0.05 vs TgRANKL+Dmab/ZOL). Student’s t-test was performed between WT and TgRANKL vehicle groups (*<0.05). Data are presented as mean values ± SD. Trabecular bone structure was assessed with BV/TV, bone volume/tissue volume; Tb.Sp, trabecular separation; Tb.N, trabecular number; Conn.Dn, connectivity density; BMD, bone mineral density. Cortical bone structure was assessed with Ct.BV/TV, cortical bone volume/tissue volume; BMV, bone marrow volume; Ct.Th, cortical thickness; Po(tot), total cortical porosity; TMD, tissue mineral density.

| **Trabecular Bone Parameters (Female)** | **WT-vehicle** | **TgRANKL-vehicle** | **TgRANKL**  **+Dmab** | **TgRANKL**  **+Dmab**  **/Discont** | **TgRANKL**  **+Dmab/ZOL** | **TgRANKL**  **+Dmab/ZOL/**  **Discont** |
| --- | --- | --- | --- | --- | --- | --- |
| BV/TV (%) | 5.88 ± 1.58 | 0.36 ± 0.24^*,b,d^ | 25.83 ± 2.83 ^a,c^ | 1.44 ± 1.45 ^b,d^ | 25.73 ± 0.82 ^a,c^ | 5.68 ± 1.81 |
| BV (mm) | 0.19 ± 0.05 | 0.02 ± 0.01^*,b,d^ | 0.88 ± 0.06^a,c^ | 0.04 ± 0.01 ^b,d^ | 0.79 ± 0.04 ^a,c^ | 0.16 ± 0.05 |
| Tb.Sp (mm) | 0.32 ± 0.02 | 1.18 ± 0.14^*,b,d^ | 0.31 ± 0.14^a,c^ | 1.14 ± 0.20 ^b,d^ | 0.52 ± 0.05 ^a,c^ | 0.58 ± 0.14 |
| Tb.N (mm^-1^) | 1.17 ± 0.28 | 0.05 ± 0.03^*,b,d^ | 3.73 ± 0.84^a,c^ | 0.23 ± 0.22 ^b,d^ | 3.19 ± 0.03 ^a,c^ | 0.98 ± 0.39 |
| Conn.Dn ( mm^-3^) | 105.1 ± 25.12 | 10.59 ± 12.40^*,b,d^ | 238.0 ± 71.12^a,c^ | 45.50 ± 46.64 ^b,d^ | 331.2 ± 118.5 ^a,c^ | 106.3 ± 62.76 |
| BMD (g/cm^3^) | 0.24± 0.02 | 0.12 ± 0.002^*,b,d^ | 0.52 ± 0.02^a,c^ | 0.15 ± 0.007 ^b,d^ | 0.54 ± 0.01 ^a,c^ | 0.26 ± 0.02 |
| **Cortical Bone Parameters (Female)** | **WT-vehicle** | **TgRANKL-vehicle** | **TgRANKL**  **+Dmab** | **TgRANKL**  **+Dmab**  **/Discont** | **TgRANKL**  **+Dmab/ZOL** | **TgRANKL**  **+Dmab/ZOL/**  **Discont** |
| Ct.BV/TV (%) | 43.22 ± 3.22 | 22.03 ± 4.26^*,b,d^ | 51.78 ± 4.20^a,c^ | 21.44 ± 2.01 ^b,d^ | 52.39 ± 1.74 ^a,c^ | 32.11 ± 5.68 |
| BMV (mm^3^) | 0.54 ± 0.04 | 1.36 ± 0.22^*,b,c,d^ | 0.51 ± 0.06^a,c^ | 1.05 ± 0.02 ^a,b,d^ | 0.57 ± 0.05 ^a,c^ | 0.96 ± 0.09 |
| Ct.Th (mm) | 0.13 ± 0.009 | 0.05 ± 0.007^*,b,d^ | 0.17 ± 0.02 ^a,c^ | 0.06 ± 0.01 ^b,d^ | 0.17 ± 0.01 ^a,c^ | 0.12 ± 0.004 |
| Po(tot) (%) | 4.81 ± 0.85 | 33.86 ± 4.60^*,b,d^ | 3.88 ± 0.46^a,c^ | 21.98 ± 9.97 ^b,d^ | 4.31 ± 1.30 ^a,c^ | 24.45 ± 4.8 |
| TMD (g/cm^3^) | 1.00 ± 0.08 | 0.35 ± 0.07^*,b,d^ | 0.84 ± 0.07^a,c^ | 0.26 ± 0.10 ^b,d^ | 0.96 ± 0.02 ^a,c^ | 0.74 ± 0.10 |

| **Trabecular Bone Parameters (Male)** | **WT-vehicle** | **TgRANKL-vehicle** | **TgRANKL**  **+Dmab** | **TgRANKL**  **+Dmab**  **/Discont** | **TgRANKL**  **+Dmab/ZOL** | **TgRANKL**  **+Dmab/ZOL/**  **Discont** |
| --- | --- | --- | --- | --- | --- | --- |
| BV/TV (%) | 16.18 ± 3.36 | 1.41 ± 1.22^*,b,d^ | 20.50 ± 8.17 ^a,c^ | 1.38 ± 1.35 ^b,d^ | 24.31 ± 3.50 ^a,c^ | 7.36 ± 2.88 |
| BV (mm) | 0.71 ± 0.18 | 0.09 ± 0.08^*,b,d^ | 0.90 ± 0.41 ^a,c^ | 0.06 ± 0.06 ^b,d^ | 1.06 ± 0.26 ^a,c^ | 0.32 ± 0.10 |
| Tb.Sp (mm) | 0.23 ± 0.02 | 1.04 ± 0.12^*,b,d^ | 0.30 ± 0.17 ^a,c^ | 0.96 ± 0.12 ^b,d^ | 0.34 ± 0.12 ^a,c^ | 0.49 ± 0.16 |
| Tb.N (mm^-1^) | 2.76 ± 0.54 | 0.19 ± 0.13^*,b,d^ | 3.51 ± 1.66 ^a,c^ | 0.20 ± 0.17 ^b,d^ | 3.88 ± 0.62 ^a,c^ | 1.20 ± 0.49 |
| Conn.Dn ( mm^-3^) | 166.2 ± 14.63 | 22.98 ± 13.68^*,b,d^ | 322.8 ± 254.4 ^a,c^ | 34.60 ± 25.29 ^b,d^ | 372.5 ± 62.44 ^a,c^ | 124.0 ± 49.59 |
| BMD (g/cm^3^) | 0.39± 0.04 | 0.12 ± 0.02^*,b,d^ | 0.44 ± 0.09 ^a,c^ | 0.18 ± 0.01 ^b,d^ | 0.52 ± 0.04 ^a,c^ | 0.26 ± 0.03 |
| **Cortical Bone Parameters (Male)** | **WT-vehicle** | **TgRANKL-vehicle** | **TgRANKL**  **+Dmab** | **TgRANKL**  **+Dmab**  **/Discont** | **TgRANKL**  **+Dmab/ZOL** | **TgRANKL**  **+Dmab/ZOL/**  **Discont** |
| Ct.BV/TV (%) | 42.01 ± 3.19 | 19.61 ± 2.16^*,b,d^ | 51.10 ± 11.93 ^a,c^ | 23.97 ± 1.74 ^b,d^ | 56.42 ± 2.81 ^a,c^ | 23.89 ± 1.93 |
| BMV (mm^3^) | 0.76 ± 0.10 | 1.61 ± 0.35^*,b,d^ | 0.68 ± 0.17 ^a,c^ | 1.32 ± 0.13 ^b,d^ | 0.65 ± 0.06 ^a,c^ | 1.37 ± 0.11 |
| Ct.Th (mm) | 0.14 ± 0.01 | 0.05 ± 0.01^*,b,d^ | 0.15 ± 0.01 ^a,c^ | 0.06 ± 0.007 ^b,d^ | 0.17 ± 0.01 ^a,c^ | 0.11 ± 0.01 |
| Po(tot) (%) | 4.65 ± 2.17 | 30.26 ± 12.0^*,b,d^ | 7.28 ± 4.27 ^a,c^ | 25.74 ± 6.18 ^b,d^ | 8.50 ± 2.12 ^a,c^ | 17.22 ± 2.13 |
| TMD (g/cm^3^) | 1.18 ± 0.15 | 0.35 ± 0.16^*,b,d^ | 1.15 ± 0.11 ^a,c^ | 0.36 ± 0.05 ^b,d^ | 1.14 ± 0.11 ^a,c^ | 0.67 ± 0.09 |

**Table S5**. Measurement of trabecular bone parameters through μCT analysis in 5^th^ lumbar spine among study groups. One-way analysis of variance (ANOVA) and Tukey post-hoc test was performed among TgRANKL groups (^a^p<0.05 vs TgRANKL-vehicle, ^b^p<0.05 vs TgRANKL+Dmab, ^c^p<0.05 vs TgRANKL+Dmab/Discont, ^d^p<0.05 vs TgRANKL+Dmab/ZOL). Student’s t-test was performed between WT and TgRANKL vehicle groups (*<0.05). Data are presented as mean values ± SD. Trabecular bone structure was assessed with BV/TV, bone volume/tissue volume; Tb.Sp, trabecular separation; Tb.N, trabecular number; Conn.Dn, connectivity density; BMD, bone mineral density. Cortical bone structure was assessed with Ct.BV/TV, cortical bone volume/tissue volume; BMV, bone marrow volume; Ct.Th, cortical thickness; Po(tot), total cortical porosity; TMD, tissue mineral density.

| **Trabecular bone Parameters** | **WT-vehicle** | **TgRANKL-vehicle** | **TgRANKL**  **+Dmab** | **TgRANKL**  **+Dmab**  **/Discont** | **TgRANKL**  **+Dmab/ZOL** | **TgRANKL**  **+Dmab/ZOL/**  **Discont** |
| --- | --- | --- | --- | --- | --- | --- |
| BV/TV (%) | 21.50 ± 3.39 | 6.82 ± 1.11^*,b,d^ | 22.72 ± 2.15 ^a,c^ | 7.69 ± 5.22 ^b,d^ | 21.39 ± 2.98 ^a,c^ | 9.82 ± 2.25 |
| BV (mm) | 0.39 ± 0.08 | 0.20 ± 0.02^*,b,d^ | 0.50 ± 007 ^a,c^ | 0.17 ± 0.10 ^b,d^ | 0.50 ± 0.16 ^a,c^ | 0.21 ± 0.04 |
| Tb.Sp (mm) | 0.22 ± 0.04 | 0.52 ± 0.04^*,b^ | 0.29 ± 0.08 ^a,c^ | 0.62 ± 0.13 ^b,d^ | 0.40 ± 0.08 ^c^ | 0.39 ± 0.03 |
| Tb.N (mm^-1^) | 4.02 ± 0.61 | 1.09 ± 0.13^*,b,d^ | 3.80 ± 0.34 ^a,c^ | 1.09 ± 0.68 ^b,d^ | 3.26 ± 0.57 ^a,c^ | 1.97 ± 0.51 |
| Conn.Dn ( mm^-3^) | 267.5 ± 46.98 | 151.6 ± 32.32^*,b,d^ | 296.2 ± 87.27 ^a,c,d^ | 137.3 ± 99.66 ^b,d^ | 417.2 ± 111.5 ^a,b,c^ | 312.0 ± 94.39 |
| BMD (g/cm^3^) | 0.44 ± 0.04 | 0.22 ± 0.01^*,b,d^ | 0.48 ± 0.02 ^a,c^ | 0.24 ± 0.06 ^b,d^ | 0.46 ± 0.04 ^a,c^ | 0.30 ± 0.03 |

| **Trabecular bone Parameters (Female)** | **WT-vehicle** | **TgRANKL-vehicle** | **TgRANKL**  **+Dmab** | **TgRANKL**  **+Dmab**  **/Discont** | **TgRANKL**  **+Dmab/ZOL** | **TgRANKL**  **+Dmab/ZOL/**  **Discont** |
| --- | --- | --- | --- | --- | --- | --- |
| BV/TV (%) | 20.11 ± 3.10 | 6.37 ± 1.04^*,b,d^ | 22.18 ± 2.07 ^a,c^ | 3.19 ± 0.54 ^b,d^ | 22.88 ± 1.27 ^a,c^ | 11.29 ± 2.29 |
| BV (mm) | 0.36 ± 0.08 | 0.19 ± 0.03^*,b,d^ | 0.51 ± 009 ^a,c^ | 0.08 ± 0.01 ^b,d^ | 0.68 ± 0.16 ^a,c^ | 0.22 ± 0.03 |
| Tb.Sp (mm) | 0.24 ± 0.03 | 0.56 ± 0.04^*,b,c,d^ | 0.37 ± 0.03 ^a,c^ | 0.74 ± 0.06 ^a,b,d^ | 0.43 ± 0.02 ^a,c^ | 0.40 ± 0.004 |
| Tb.N (mm^-1^) | 3.78 ± 0.57 | 1.05 ± 0.18^*,b,d^ | 3.59 ± 0.29 ^a,c^ | 0.50 ± 0.06 ^b,d^ | 3.70 ± 0.15 ^a,c^ | 2.42 ± 0.43 |
| Conn.Dn ( mm^-3^) | 276.9 ± 53.60 | 142.2 ± 89.36^*,d^ | 246.5 ± 22.24 ^c,d^ | 70.0 ± 11.64 ^b,d^ | 384.5 ± 41.95 ^a,b,c^ | 399.3 ± 37.73 |
| BMD (g/cm^3^) | 0.43 ± 0.04 | 0.21 ± 0.009^*,b,d^ | 0.47 ± 0.02 ^a,c^ | 0.18 ± 0.007 ^b,d^ | 0.50 ± 0.02 ^a,c^ | 0.31 ± 0.03 |

| **Trabecular bone Parameters (Male)** | **WT-vehicle** | **TgRANKL-vehicle** | **TgRANKL**  **+Dmab** | **TgRANKL**  **+Dmab**  **/Discont** | **TgRANKL**  **+Dmab/ZOL** | **TgRANKL**  **+Dmab/ZOL/**  **Discont** |
| --- | --- | --- | --- | --- | --- | --- |
| BV/TV (%) | 24.76 ± 0.46 | 7.02 ± 1.16^*,b,d^ | 23.09 ± 2.31 ^a,c^ | 11.44 ± 4.14 ^b,d^ | 20.80 ± 3.37 ^a,c^ | 8.84 ± 2.00 |
| BV (mm) | 0.46 ± 0.04 | 0.21 ± 0.02^*,b,d^ | 0.50 ± 006 ^a,c^ | 0.24 ± 0.09 ^b,d^ | 0.43 ± 0.12 ^a,c^ | 0.21 ± 0.05 |
| Tb.Sp (mm) | 0.18 ± 0.01 | 0.51 ± 0.04^*,b,^ | 0.25 ± 0.07 ^a,c,d^ | 0.53 ± 0.09 ^b,d^ | 0.40 ± 0.09 ^b,c^ | 0.39 ± 0.04 |
| Tb.N (mm^-1^) | 4.58 ± 0.02 | 1.10 ± 0.12^*,b,d^ | 3.94 ± 0.30 ^a,c,d^ | 1.58 ± 0.55 ^b,d^ | 3.08 ± 0.60 ^a,b,c^ | 1.66 ± 0.28 |
| Conn.Dn ( mm^-3^) | 245.5 ± 16.78 | 138.8 ± 17.62^*,b,d^ | 329.3 ± 100.6 ^a^ | 197.4 ± 100.9 ^d^ | 430.2 ± 132.2 ^a,c^ | 253.9 ± 66.50 |
| BMD (g/cm^3^) | 0.48 ± 0.01 | 0.23 ± 0.008^*,b,c,d^ | 0.49 ± 0.02 ^a,c,e^ | 0.29 ± 0.05 ^a,b,d^ | 0.45 ± 0.03 ^a,c^ | 0.30 ± 0.05 |

**Table S6**. Measurement of Adipose volume fraction as determined by μCT and osmium tetroxide staining of distal femurs from WT, TgRANKL, TgRANKL+Dmab, TgRANKL+Dmab/Discont groups . One-way analysis of variance (ANOVA) and Tukey post-hoc test was performed among TgRANKL groups (^a^p<0.05 vs TgRANKL-vehicle, ^b^p<0.05 vs TgRANKL+Dmab, ^c^p<0.05 vs TgRANKL+Dmab/Discont). Student’s t-test was performed between WT and TgRANKL vehicle groups (*<0.05). Data are presented as mean values ± SD.; Ad.V/Ma.V, Adipose volume/Marrow volume.

| **Trabecular bone Parameters** | **WT-vehicle** | **TgRANKL-vehicle** | **TgRANKL**  **+Dmab** | **TgRANKL**  **+Dmab**  **/Discont** |
| --- | --- | --- | --- | --- |
| Metaphysis Ad.V/Ma.V (%) | 0.42 ± 0.49 | 61.95 ± 9.39^*,b,c^ | 1.17 ± 1.19 ^a,c^ | 9.95 ± 2.68 ^a,b^ |
| Diaphysis Ad.V/Ma.V (%) | 0.04 ± 0.05 | 43.77 ± 13.35^*,b,c^ | 0.31 ± 0.24 ^a^ | 7.31 ± 3.69 ^a^ |
